# A Modified Polynomial Hysteretic Model for Asymmetric Vertical Hysteretic Behavior of Inclined Rubber Bearings

**DOI:** 10.3390/polym18060686

**Published:** 2026-03-12

**Authors:** Zhixun Li, Yangyang Chen, Zhongling Xiao, Bo Liu

**Affiliations:** 1 MOE Key Laboratory of Earthquake Resistance, Earthquake Mitigation and Structural Safety, Guangzhou University, Guangzhou 510405, China; 2 Guangdong Provincial Key Laboratory of Earthquake Engineering and Applied Technology, Guangzhou University, Guangzhou 510405, China; 3 Guangzhou City Construction & Development Co., Ltd., Guangzhou 510623, China

**Keywords:** inclined rubber bearing, modified nonlinear hysteresis polynomial model, hysteresis weighting factor, hysteresis performance

## Abstract

In the field of mechanical engineering, inclined rubber bearings reduce vertical stiffness through tilted arrangement to effectively isolate environmental vibrations. When applied to large-scale structural engineering, however, further attention must be paid to their vertical hysteretic performance under large deformation, so as to provide a basis for three-dimensional seismic isolation analysis of structures. Traditional seismic design often simplifies the vertical constitutive model of bearings as linear, while tests have shown that the vertical behavior of inclined rubber bearings exhibits significant asymmetric hysteretic characteristics, which cannot be accurately described by existing symmetric constitutive models. In this paper, vertical performance tests are further conducted on inclined rubber bearing specimens, and a modified hysteretic polynomial model is proposed to adapt it to the theoretical description of asymmetric vertical hysteretic behavior of inclined rubber bearings. Through parameter modification, device testing, and comparative analysis of results, the accuracy and effectiveness of the model are verified, providing a theoretical basis for its engineering application.

## 1. Introduction

In the field of mechanical engineering, inclined rubber bearings reduce the overall vertical stiffness by being tilted at a specific angle, thereby effectively isolating environmental vibrations. This type of bearing has also been explored for application in bridge engineering to mitigate train-induced vibrations. However, as bridges are large-scale structures, the bearings must be evaluated for their potential to undergo significant vertical deformation under tri-directional seismic excitation. It is therefore essential to further investigate their vertical hysteretic performance under such large-deformation conditions. However, in structural design, traditional seismic isolation bearings typically focus solely on their horizontal mechanical performance under the assumption that vertical deformation remains negligible, even when horizontal deformation increases significantly during major earthquakes. Clearly, this assumption does not hold for inclined rubber bearings. Therefore, it is necessary to establish a constitutive model that is capable of accurately describing their vertical hysteretic behavior, thereby providing a theoretical basis for their further application in engineering practice.

Previous studies have predominantly simplified the vertical constitutive model of seismic isolation bearings as linear or piecewise linear models [1,2,3]. However, studies on the vertical mechanical behavior of seismic isolation bearings [4,5,6,7,8] indicate that their vertical stiffness depends on multiple factors, including contact pressure, shear deformation, and rubber layer thickness. This reveals that simplifying the vertical stiffness as linear is inadequate, as it inherently exhibits nonlinear characteristics. Gupta et al. [9] conducted performance tests on high-damping rubber bearings, and Mavronicola et al. [10] performed performance tests on lead rubber bearings, which have also confirmed this finding. In the seismic design of tall base-isolated structures, under near-fault ground motions, or in multi-functional vibration isolation systems, the results calculated using linear stiffness assumptions can differ substantially from those obtained when considering nonlinear stiffness.

However, the aforementioned research achievements predominantly focus on constitutive analytical models for the horizontal behavior of seismic isolation bearings and experimental investigations into their vertical mechanical properties. Current studies on vertical constitutive models are largely limited to elastic models. Extensive experimental data on the vertical mechanical performance of isolation bearings reveal that their vertical hysteretic loops are asymmetric curves [11,12,13,14], making the accurate description of these curves considerably more challenging. Consistent with the findings of Chisari et al. [15], the classical Bouc–Wen model, a typical phenomenological hysteretic model, adopts a core characterization logic of accomplishing parameter calibration by using experimental data to accurately fit macroscopic hysteretic behavior, without delving into the microscopic constitutive mechanisms of materials or analyzing the force transfer laws inside bearings. However, existing relevant studies have mainly focused on the characterization and identification of the symmetric hysteretic properties of rubber bearings [16,17,18,19]. Nevertheless, scholars such as Wu, Yi, and Wang et al. [20,21,22,23,24] have explicitly pointed out that the classical Bouc–Wen model produces point-symmetric hysteretic loops, making it incapable of effectively capturing the vertical asymmetric hysteretic characteristics of rubber seismic isolation bearings. Hwang et al. [25] proposed a phenomenological model that can accurately characterize the horizontal hysteretic behavior of high-damping elastomeric bearings, yet this model also fails to describe the asymmetric vertical hysteresis of rubber bearings. Zhang et al. [26], Lyu Yang et al. [27], and Park et al. [28] conducted experimental studies on lead-core thick rubber bearings, magnetorheological elastomer bearings, high-damping rubber bearings, and other types of bearings, which have also confirmed this conclusion. Moreover, Zhang et al. [29] identify more complex asymmetric features in the vertical hysteresis of three-dimensional isolation bearings, providing critical experimental evidence for subsequent model refinements.

In summary, existing research still lacks a phenomenological theoretical model that is capable of accurately describing the vertical constitutive relationship of inclined rubber bearings. The polynomial hysteresis model, as a theoretical framework with potential for improvement, was successfully applied by Gjorgjiev and Garevski [30] to describe the horizontal mechanical behavior of rubber bearings. However, this model does not account for asymmetric constitutive scenarios. To address this limitation, this study proposes a modified hysteretic polynomial model, extending it to characterize the asymmetric vertical hysteretic behavior of inclined rubber bearings. Through parameter modification, device testing, and comparative analysis of results, the validity of the model is systematically verified.

## 2. Hysteretic Polynomial Model

Gjorgjiev and Garevski proposed a straightforward hysteretic polynomial model for rubber bearings, which is hereinafter referred to as the original polynomial model. This model employs polynomial functions and introduces eight additional parameters, determined through biaxial testing. It is capable of simulating the horizontal mechanical behavior of natural rubber bearings under both small and large deformation conditions. The model comprises three distinct phases: a linear-elastic phase, a post-yield loading phase, and a post-yield unloading phase. In the pre-yield phase, corresponding to the linear-elastic regime under small deformations, the relationship is formulated as

(1) $$F=K_{1}\cdot D$$

where *F* is the horizontal load, *D* is the horizontal displacement, and *K*_1_ is the initial stiffness of the rubber bearing. This pre-yield phase also represents the transition stage of the model from unloading to reloading. The input parameters for this phase are the elastic stiffness *K*_1_ and the yield displacement *D_γ_* of the bearing, both of which are determined from the bearing test results.

When the bearing enters the post-yield loading phase, the force–displacement relationship is defined by a polynomial function as

(2) $$F_{i}=F_{0m}^{i}+a_{1}D_{i}+a_{2}D_{i}^{2}+a_{3}D_{i}^{3}+\ldots$$

where *F_i_* is the horizontal load, *D_i_* is the horizontal displacement, and $a_{1}$, $a_{2}$, and $a_{3}$ are the parameters of the polynomial function. In Equation (2), the polynomial term $F_{0m}^{i}\equiv F_{0}^{i}$ is a variable that is dependent on the current state, while the polynomial term $F_{0m}^{i}$ is a variable associated with the loading history. Concurrently, a load-related quantity $F_{0}^{i}$, defined as a linear variation, is determined by the following equation:

(3) $$F_{0}^{i}=\left\{\begin{array}{l} F_{0}^{\min}\;\;\;\;\;\;\;\;\;\;\;\;\;\;\;\;\;\;\;\;\;\;\;\;\;\;\;\;\;\;\;\;\;\;\;\;\;\;\;\;\;\;\;\;\;\;\;\;\;\;\;,D_{i}^{kp}<D_{0}^{\min}\\ F_{0}^{\min}+(F_{0}^{\max}-F_{0}^{\min})\frac{D_{i}^{kp}-D_{0}^{\min}}{D_{0}^{\max}-D_{0}^{\min}}\;\;\;\;\;\;\;\;\;\;\;,D_{0}^{\min}<D_{i}^{kp}<D_{0}^{\max}\\ F_{0}^{\max}\;\;\;\;\;\;\;\;\;\;\;\;\;\;\;\;\;\;\;\;\;\;\;\;\;\;\;\;\;\;\;\;\;\;\;\;\;\;\;\;\;\;\;\;\;\;\;\;\;\;\;,D_{i}^{kp}>D_{0}^{\max} \end{array}\right.$$

where

(4) $$D_{i}^{kp}=\max(D_{i},D^{kp})$$

When the bearing enters the post-yield unloading phase, the force–displacement relationship is also described by the polynomial function given in Equation (2). To capture the variation in mechanical properties of different rubber bearings during unloading, the parameter $F_{0m}^{i}$ in Equation (2) is expressed as

(5) $$F_{0m}^{i}=F_{i}^{0}(2\cdot kF_{i}^{0}-1)$$

where $kF_{i}^{0}$ is the attenuation parameter and is expressed by the following equation:

(6) $$kF_{i}^{0}=\frac{\exp(eD_{i}^{kp}\cdot \frac{D_{i}}{D_{i}^{kp}})-1}{\exp(eD_{i}^{kp})-1}$$

The input parameters for this phase are the displacements $D_{e}^{\max}$ and $D_{e}^{\min}$.

However, this polynomial model exhibits limitations in characterizing the hysteretic behavior of rubber bearings. It fails to adequately capture the softening behavior observed in rubber bearings under large deformations, making it difficult to accurately characterize their hysteretic response. Notably, the model is particularly incapable of accurately representing asymmetric hysteretic characteristics. For inclined rubber bearings, which serve as vertical isolation devices, their vertical mechanical behavior often demonstrates pronounced asymmetry.

## 3. Modified Hysteretic Polynomial Model for Inclined Rubber Bearings

To address the issues mentioned above, this study proposes a modified hysteretic polynomial model by introducing a hysteretic weighting coefficient, n, and an optimization parameter, m, based on the original polynomial model. Given the absence of a distinct yield point in the vertical response curve of the isolation device, the modified hysteretic polynomial model only incorporates two phases for characterizing its vertical performance: a loading phase and an unloading phase. The model adopts a displacement-controlled criterion to distinguish between these states: it is identified as loading when the vertical displacement increases ($D_{i}>D_{i-1}$) and as unloading when the vertical displacement decreases ($D_{i}<D_{i-1}$). The force–displacement relationship of the model is defined by the following polynomial function:

(7) $$F_{i}=nF_{0}^{i}+a_{1}D_{i}+a_{2}D_{i}^{2}+a_{3}D_{i}^{3}+\ldots$$

where $F_{i}$ is the vertical force, $D_{i}$ is the vertical displacement, and *n*, $a_{1}$, $a_{2}$, and $a_{3}$ are the hysteretic weighting parameter, linear parameter, quadratic parameter, and cubic parameter of the polynomial, respectively. Optimal values for these parameters can be obtained by fitting the experimental data using the nonlinear least squares method. Therefore, the proposed model successfully addresses the limitation of the original polynomial model in accurately describing asymmetric hysteretic behavior, thereby enabling effective simulation of the vertical nonlinear hysteretic mechanical response of inclined rubber bearings.

When the seismic isolation bearing is in the loading phase, the parameter in the model is set as $F_{0}^{i}=F_{0z}^{i}$. The model describes the hysteretic characteristics of the bearing through $F_{0}^{i}$, which is determined based on experimental data and the current vertical deformation. The determination of $F_{0z}^{i}$ can be formulated as

(8) $${F_{0z}}^{i}={F_{0\min}}^{i}+(F_{0\max}-{F_{0\min}}^{i})(\frac{D_{i}^{kp}-{D_{0\min}}^{i}}{D_{0\max}-{D_{0\min}}^{i}})$$

where $D_{i}^{kp}$ is an introduced variable in the model. During the loading process, $D_{i}^{kp}$ equals $D_{i}$. The vertical force $F_{0\max}$ and the vertical displacement $D_{0\max}$ serve as input parameters of the model, which are determined from experimental data.

During the unloading phase, the parameter $F_{0z}^{i}$ can also be expressed by Equation (8), where

(9) $$D_{i}^{kp}=\max(D_{i-1},{D_{i-1}}^{kp})$$

**Figure 1:**
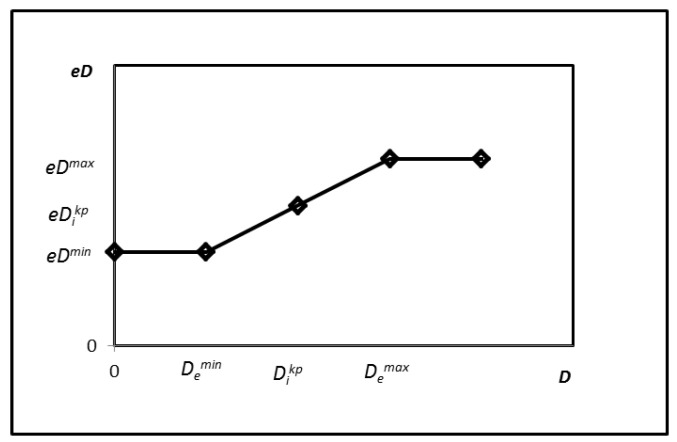
The $eD_{i}^{kp}-D_{i}^{kp}$ relationship.

During the unloading phase, $D_{i}^{kp}$ is the vertical displacement of the bearing at the initial unloading instant. A decay parameter, $dF_{0}^{i}$, is introduced in the unloading phase to characterize the variations in the vertical stiffness and performance of the seismic isolation device during the unloading process. The unloading rate of the model can be regulated by this decay parameter, which is expressed by Equations (10) and (11) as follows:

(10) $$F_{0}^{i}={\left(dF_{0}^{i}\right)}^{m}\cdot {F_{0z}}^{i}$$

(11) $$dF_{0}^{i}=\frac{\exp(eD_{i}^{kp}\frac{D_{i}}{D_{i}^{kp}})-1}{\exp(eD_{i}^{kp})-1}$$

where *e* is the natural constant (*e* = 2.71828…), and *m* is a control parameter that serves to adjust the unloading rate. Parameter $F_{0}^{i}$ is derived from Equation (10). Its value depends on the current vertical displacement and the historical loading path, thereby governing the hysteretic characteristics of the model. The term $dF_{0}^{i}$ in Equation (11) comprises three parameters ($eD_{i}^{kp}$, $D_{i}$, $D_{i}^{kp}$), all of which are dependent on the current vertical displacement and the historical loading path. The parameter $eD_{i}^{kp}$ is derived from Equation (12) and is directly dependent on $D_{i}^{kp}$. Figure 1 illustrates the relationship between $eD_{i}^{kp}$ and $D_{i}^{kp}$. In Equations (13) and (14), $eD_{\min}$ and $eD_{\max}$ exhibit a natural logarithmic relationship with $D_{e}^{\min}$ and $D_{e}^{\max}$, respectively.

(12) $$eD_{i}^{kp}=eD_{\min}-(eD_{\max}-eD_{\min})\frac{D_{i}^{kp}-D_{e}^{\min}}{D_{e}^{\max}-D_{e}^{\min}}$$

(13) $$eD_{\min}=\ln(D_{e}^{\min})$$

(14) $$eD_{\max}=\ln(D_{e}^{\max})$$

During the unloading process, $D_{i}^{kp}$ corresponds to a constant value representing the maximum vertical displacement attained in the loading phase. The current displacement control parameter $D_{i}$ is bounded within the range [0, $D_{i}^{kp}$].

(15) $$\left\{\begin{array}{ccc} D_{i}=0 & \Rightarrow & dF_{0}^{i}=0\\ D_{i}=D_{i}^{kp} & \Rightarrow & dF_{0}^{i}=1 \end{array}\right.$$

(16) $$\left\{\begin{array}{ccc} D_{i}=0 & \Rightarrow & F_{0}^{i}=0\\ D_{i}=D_{i}^{kp} & \Rightarrow & F_{0}^{i}={F_{0z}}^{i} \end{array}\right.$$

By combining Equations (11), (15) and (16), it can be concluded that the decay parameter $dF_{0}^{i}$ is bounded within the interval [0, 1]. Correspondingly, the model parameter $F_{0}^{i}$ satisfies the condition that $F_{0}^{i}=\;\;0$ when the bearing experiences zero displacement, and $F_{0}^{i}=\;\;F_{0z}^{i}$ during the initial phase of unloading.

**Figure 2:**
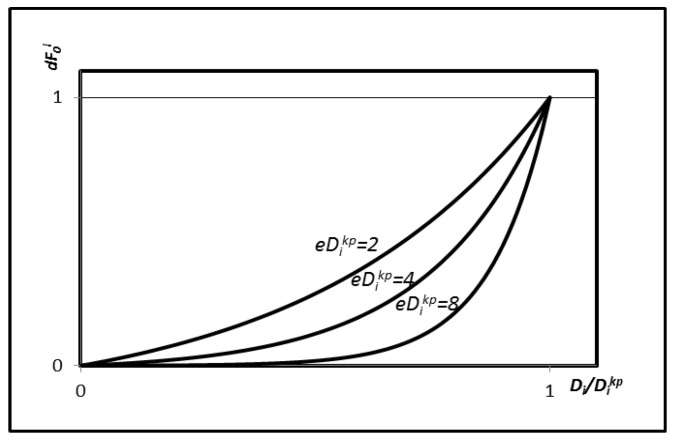
The value of $dF_{0}^{i}$ in the post-yield state at unloading.

To further investigate the relationship between $eD_{i}^{kp}$ and the vertical displacement of the bearing, three specific values of $eD_{i}^{kp}$ are considered in this study, 2, 4, and 8, as presented in Figure 2. It can be observed from the figure that a larger value of $eD_{i}^{kp}$ results in a faster and sharper unloading rate. Comparison among the three values indicates that the decay parameter $dF_{0}^{i}$ consistently satisfies $dF_{0}^{i}\in [0,1]$ during both the initial and final stages of unloading.

The parameter *m* is the optimization parameter of the modified model. By varying *m*, the unloading rate of the model can be controlled. A larger value of *m* corresponds to a faster unloading rate, while a smaller value results in a slower unloading rate. The parameter *n* is the hysteretic weighting parameter of the modified model. Optimizing *n* enables the model to achieve a better fit to the experimental data.

The modified polynomial model features a simple expression form, low computational cost and an inherent consideration of the effects of the loading history. Compared with the traditional Bouc–Wen model and the original polynomial hysteretic model, this proposed model breaks through their inherent limitations in characterizing asymmetric hysteresis and is capable of accurately simulating the more complex characteristics of vertical asymmetric hysteretic loops exhibited by inclined rubber bearings, thus achieving a significant improvement in its adaptability and engineering practicability.

## 4. Verification of the Modified Hysteretic Polynomial Model for Inclined Rubber Bearings

### 4.1. Analysis of Vertical Mechanical Performance Experiment Results

**Figure 3:**
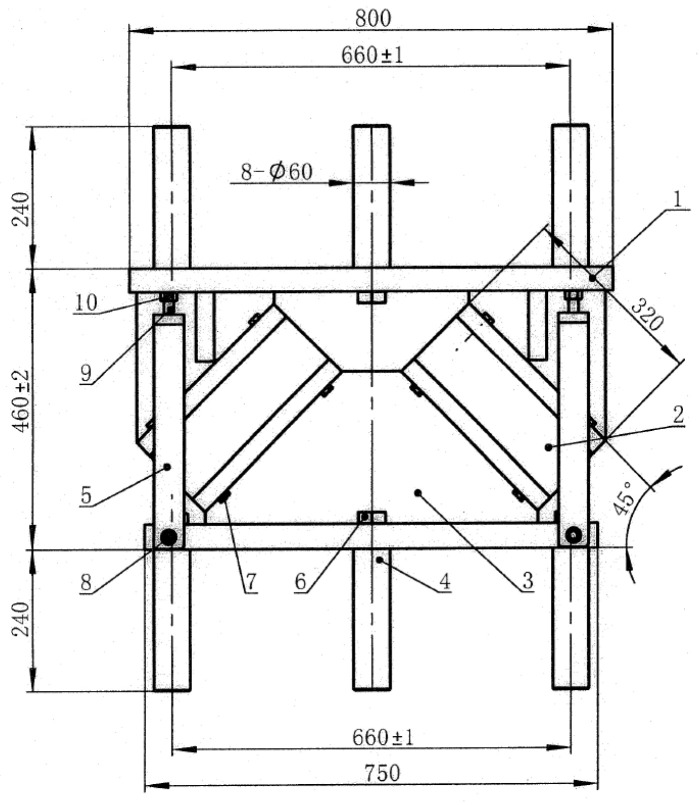
Experimental model of inclined rubber bearing.

**Figure 4:**
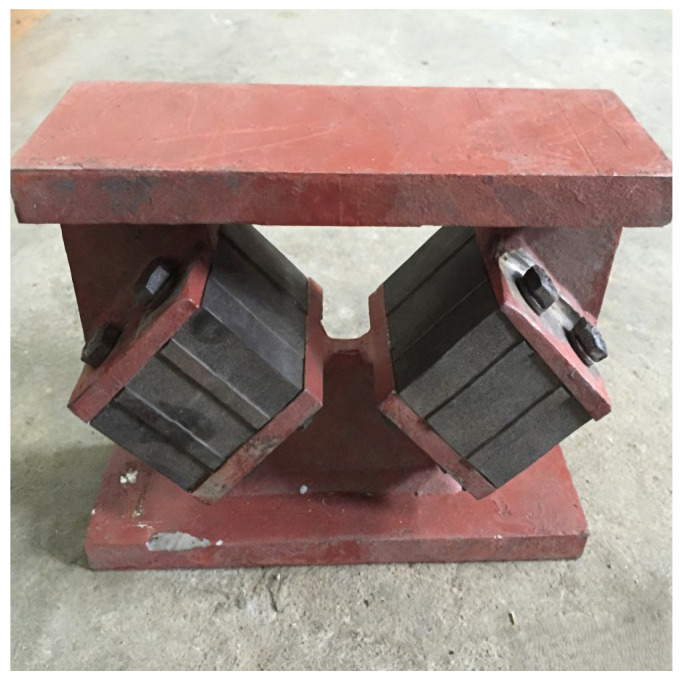
Experimental specimen of inclined rubber bearing.

**Figure 5:**
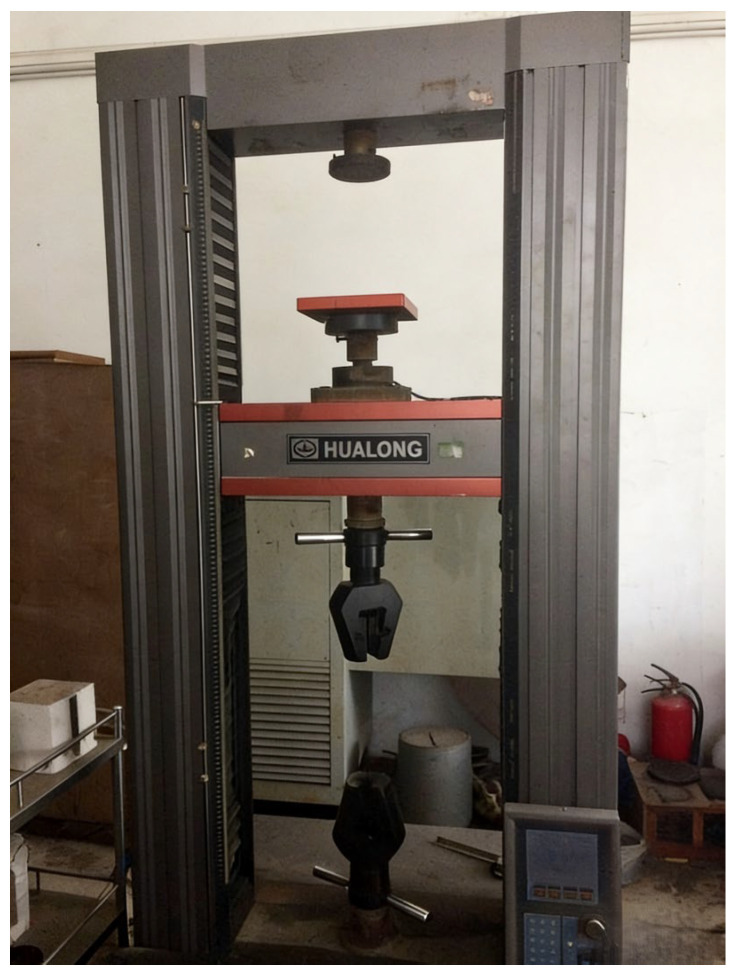
Universal testing machine.

To verify the rationality of the proposed modified model, due to the constraints of test conditions and experimental costs, this study took the experimental prototype of the inclined rubber bearing as a reference. In accordance with the Buckingham π theorem, the dimensionless products of the control parameters were derived through dimensional analysis, and the similarity ratios of each control parameter were determined on this basis. Eventually, four test specimens of the same specification were fabricated at a scaling ratio of 1:5.5. The experimental prototype and the fabricated test specimens of the inclined rubber bearing are shown in Figure 3 and Figure 4, respectively. Furthermore, vertical cyclic loading experiments were conducted on a universal testing machine, as shown in Figure 5. Five vertical basic performance experiments under different loading cases were performed for each bearing: the experimental specimens were subjected to loading and unloading at loading rates of 1, 5, 10, 20, and 30 mm/s respectively, with the vertical load ranging from 23.98 kN to 73.57 kN for cyclic loading and unloading. Details of each loading case are presented in Table 1.

**Figure 6:**
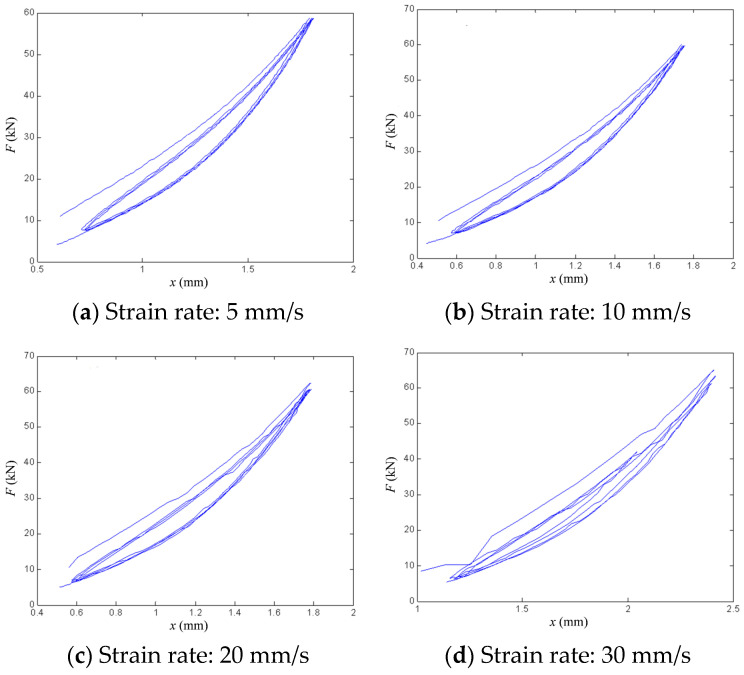
Vertical force–displacement curves of bearing 1# under different strain rates.

**Figure 7:**
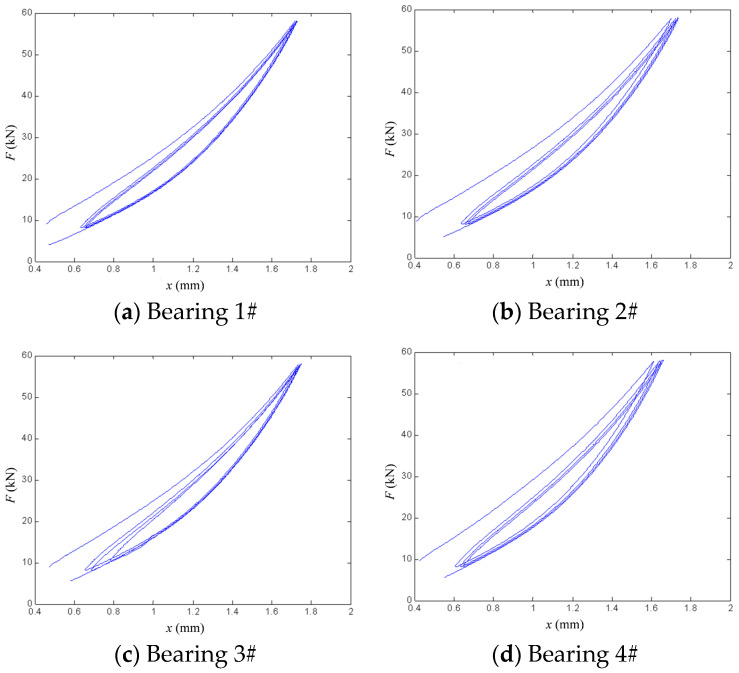
Vertical force–displacement curve of bearing 1# under strain rate of 1 mm/s.

The vertical load–displacement curves obtained from the vertical performance experiment are presented in Figure 6 and Figure 7. Figure 6 shows the experimental curves for bearing 1# under strain rates of 5, 10, 20, and 30 mm/s. Figure 7 presents the experimental curves for each bearing tested at a strain rate of 1 mm/s. As observed in the results, the vertical stiffness exhibits a marginally increasing trend with growing vertical displacement, although the extent of this increase is limited. Correspondingly, the hysteretic curves remain predominantly linear. Moreover, all four inclined bearings demonstrate notable energy dissipation, as evidenced by clearly discernible hysteretic loops in their force–displacement responses.

### 4.2. Comparative Analysis of the Modified Hysteretic Polynomial Model for Different Experiment Results

**Figure 8:**
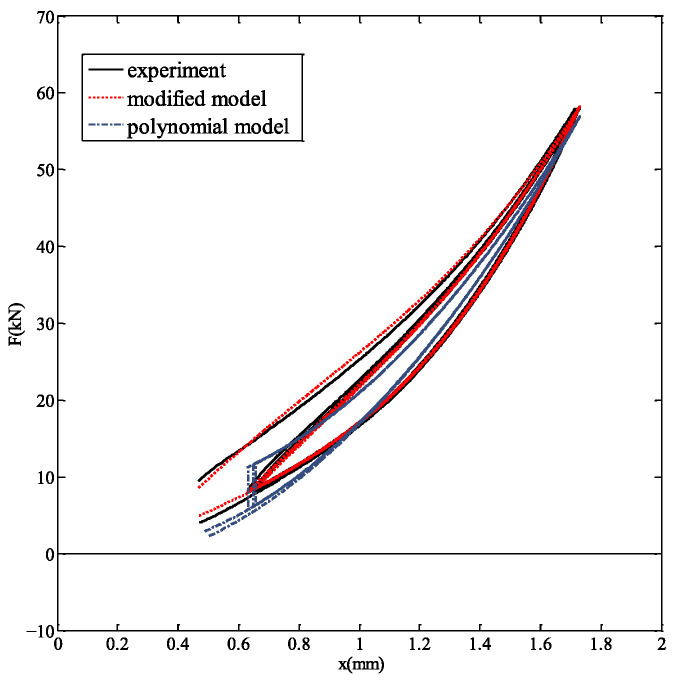
Comparison of test data with simulation results for bearing 1# under strain rate of 1 mm/s.

Based on the experimental data for inclined rubber bearings, a comparative analysis is conducted between the modified hysteretic polynomial model and the experimental results to validate the feasibility of the modified model. The experimental data for bearing 1# at a loading rate of 1 mm/s were fitted using both the modified hysteretic polynomial model and the original polynomial model. The comparative results for bearing 1# at the 1 mm/s loading rate are presented in Figure 8. Table 2 presents the error comparison between the experimental data and the simulated vertical forces obtained from the original polynomial model and the modified hysteretic polynomial model.

As shown in Figure 8, the modified hysteretic polynomial model demonstrates significantly better agreement with the experimental data than the original polynomial model. Table 2 presents the evaluation indicators of both models. The modified hysteretic polynomial model achieves a maximum absolute error ${\left|e\right|}_{\max}$ of 0.9635 kN and a goodness of fit *R*^2^ of 0.9904, outperforming the original polynomial model in both measures.

**Figure 9:**
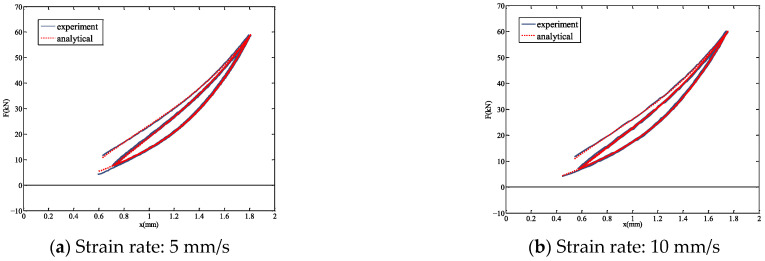

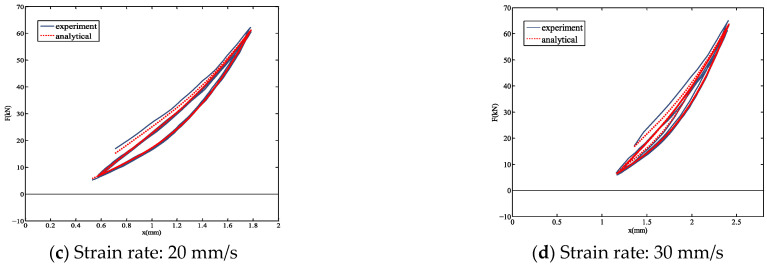
Comparative analysis between test and analytical model for 1# bearing.

To account for the influence of different loading rates, the experimental data for bearing 1# under various strain rates were compared with the results of the modified hysteretic polynomial model. The comparative curves between the simulations and the experimental data at strain rates of 5, 10, 20, and 30 mm/s are shown in Figure 9a–d, respectively. Under slower strain rates (5 and 10 mm/s), the modified hysteretic polynomial model closely aligns with the experimental values. In contrast, at higher strain rates (20 and 30 mm/s), the model exhibits greater deviation from the experimental data. Specifically, during the initial loading ascent at 30 mm/s, the fitting performance is notably less accurate. Overall, the precision of the fitting results decreases as the strain rate increases, which can be attributed to the reduced accuracy of data acquisition during high-rate experiments. Furthermore, Figure 9d reveals that even at a strain rate of 30 mm/s, clearly discernible hysteretic loops are present, indicating that the modified hysteretic polynomial model can effectively mitigate the influence of experimental errors on the bearing response. Table 3 lists the values of the parameters in Equation (7) for bearing 1# under five different strain rates.

Table 4 presents a comparison of the fitting errors for bearing 1# under each strain rate. The goodness of fit *R*^2^ values for all five cases are notably high, demonstrating the feasibility of the parameter identification method. At strain rates of 1, 5, and 10 mm/s, the errors remain relatively low, indicating excellent fitting performance. In contrast, at strain rates of 20 and 30 mm/s, the errors are comparatively higher. It can be observed that the fitting accuracy exhibits a decreasing trend as the loading rate increases.

**Figure 10:**
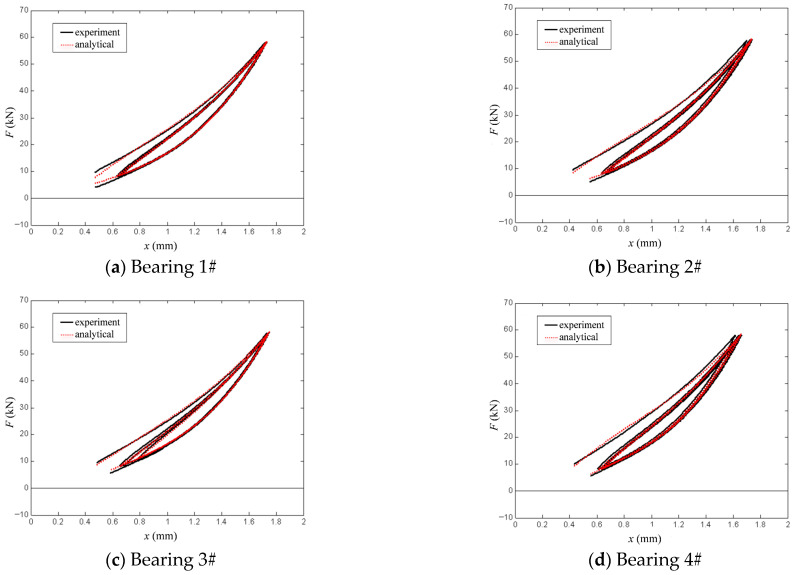
Comparison of simulation fitting errors under strain rate of 1 mm/s.

Figure 10 presents a comparison between the experimental data for the four bearings and the calculated results from the modified hysteretic polynomial model at a strain rate of 1 mm/s. All four bearings exhibit relatively plump hysteretic loops. Table 5 presents the parameter values of the modified model for each bearing at a loading rate of 1 mm/s. It can be observed that parameter *n* and optimization parameter *m* exhibit an inverse proportional relationship, and they synergistically control the hysteretic curves of the bearings, specifically by influencing the nonlinear stiffness and nonlinear damping.

Table 6 presents a comparison of the fitting errors for each bearing at the strain rate of 1 mm/s. It can be observed that the goodness of fit *R*^2^ values for all bearings exceed 0.98, with relatively low mean absolute errors ${\left|e\right|}_{mean}$ and root mean square errors $e_{MSE}$. Furthermore, the maximum absolute error ${\left|e\right|}_{\max}$ is controlled within the range of 1 to 3 kN. The results indicate that the modified hysteretic polynomial model can fit the experimental data well, with high fitting accuracy.

**Figure 11:**
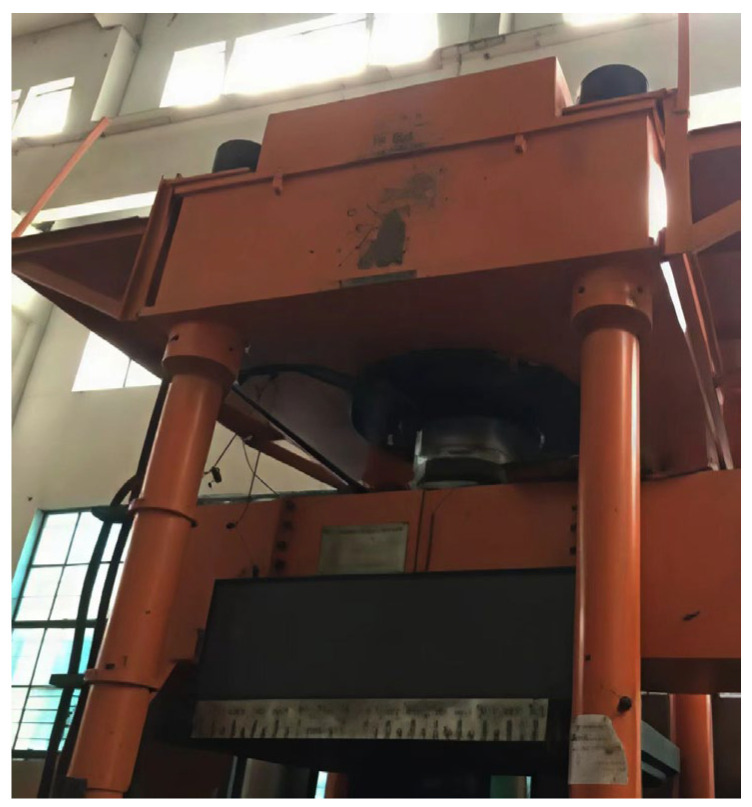
Electrohydraulic servo-compression shear test system.

**Figure 12:**
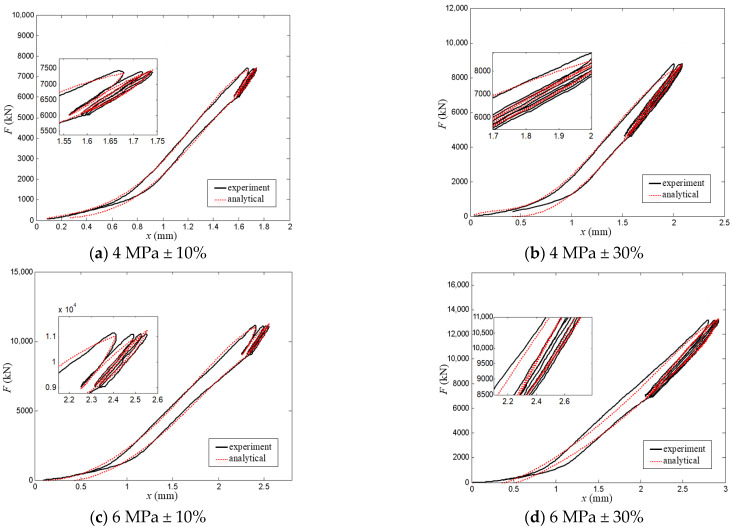
Comparison of test data and simulation results for thick laminated rubber bearing.

To further verify the engineering adaptability and wide applicability of the proposed modified hysteretic polynomial model, vertical compression tests on a thick laminated rubber bearing were conducted in this study. The external dimensions of the thick laminated rubber bearing are 1300 mm × 1300 mm × 248 mm, and it is composed of an upper connection plate, an upper embedded plate, a bearing core, a lower embedded plate and a lower connection plate. The schematic diagram of the loading system is shown in Figure 11. A total of four test contact pressure cases were designed for the tests, with the detailed test parameters being listed in Table 7. Furthermore, the experimentally measured results were compared and analyzed with the calculated results of the modified hysteretic polynomial model with identified parameters, and the comparison results are presented in Figure 12.

It can be seen from Figure 12 that the fitting results of the proposed modified hysteretic polynomial model in this study are in good overall agreement with the experimental data, and the model can clearly reflect the plump characteristics of the hysteretic loops of the thick laminated rubber bearing. Table 8 lists the model parameter values of the thick laminated rubber bearing under different controlled load cases, while Table 9 presents the comparison results of the model fitting errors under each controlled load case. The results show that the goodness of fit of the model remains at a high level under all four load cases, which fully verifies the effectiveness and reliability of the modified hysteretic polynomial model proposed in this study. It also demonstrates that the model is not merely applicable to inclined rubber bearings but also possesses a favorable ability to characterize the hysteretic behavior of thick laminated rubber bearings, thus reflecting the wide engineering applicability of the model.

## 5. Conclusions

In this study, vertical performance experiments were conducted on inclined rubber bearings under different levels of displacement control with reciprocating loads to determine their fundamental vertical properties. A modified hysteretic polynomial model was established to fit the vertical experimental curves of the inclined rubber bearings. Subsequently, the modified hysteretic polynomial model and the experimental results were compared and analyzed to verify the model’s validity and fitting performance. The principal findings are as follows:

(1) From the vertical basic-performance experimental curves, it can be observed that with the continuous increase in vertical deformation, the vertical stiffness of the bearings gradually increases, but the range of increase is very small. The inclined rubber bearings exhibit significant energy dissipation behavior; specifically, the force–displacement curves feature relatively distinct hysteretic loops.
(2) A modified hysteretic polynomial model based on inclined rubber bearings was established to simulate the vertical experimental results of the bearings. Through comparative analysis, it is found that at a strain rate of 1 mm/s for bearing 1#, the modified hysteretic polynomial model exhibits significantly better fitting performance than the original polynomial model.
(3) Based on the comparison of fitting results and analysis of simulation errors for bearing 1# under different strain rates, it is concluded that the fitting performance under slow strain rates is significantly better than that under fast strain rates. This is attributed to the fact that the higher the strain rate is, the lower the accuracy of the data collected during the experiment is. Notably, obvious hysteretic loops can still be observed at a strain rate of 30 mm/s, indicating that the modified hysteretic polynomial model can effectively mitigate the influence of experimental errors on the bearing.
(4) Based on the comparison of fitting results and simulation error analysis for all four bearings at a strain rate of 1 mm/s, it is concluded that the experimental curves of all four bearings can be fitted well by the modified hysteretic polynomial model, and they exhibit relatively high fitting accuracy.
(5) Based on the fitting and error analysis of the vertical compression test results for thick laminated rubber bearings under different test contact pressure cases, it is concluded that the modified hysteretic polynomial model achieves good fitting performance for the test data for such bearings, and it also possesses a good capability to characterize the hysteretic behavior of thick laminated rubber bearings, which fully reflects the remarkable generalization ability and wide engineering applicability of the model.

## Figures and Tables

**Table 1:** Vertical mechanical performance test conditions for inclined rubber bearing.

No.	Experimental Bearing Specimen	Strain Rate (mm/s)	Cycles	Vertical Load (kN)
1	1#, 2#, 3#, 4#	1	4	23.98–73.57
2		5		
3		10		
4		20		
5		30		

**Table 2:** Comparison of simulation fitting errors.

Evaluation Indicator	Baseline Polynomial Model	Modified Polynomial Model
Mean square error $e_{MSE}$ (kN)	5.8658	0.1185
Mean absolute error ${\left|e\right|}_{mean}$ (kN)	1.6052	0.2705
Maximum absolute error ${\left|e\right|}_{\max}$ (kN)	9.0609	0.9635
Goodness of fit $R^{2}$	0.9328	0.9904

**Table 3:** Modified polynomial model parameters for bearing 1#.

No.	Strain Rate/(mm/s)	*n*	*a*_1_	*a*_2_	*a*_3_	*a*_4_	*a*_5_
1	1	1.052	−6.448	23.049	−45.388	28.985	−5.503
2	5	0.761	−9.207	44.789	−56.880	29.329	−4.800
3	10	0.761	−7.275	45.509	−67.181	39.782	−7.646
4	20	0.733	−10.843	58.689	−83.005	48.126	−9.301
5	30	0.379	−43.782	98.687	−76.706	27.775	−3.592

**Table 4:** Simulation fitting errors for bearing 1#.

Evaluation Indicator	Strain Rate				
	1 mm/s	5 mm/s	10 mm/s	20 mm/s	30 mm/s
Mean square error $e_{MSE}$ (kN)	0.1185	0.1247	0.1335	0.4274	1.1492
Mean absolute error ${\left|e\right|}_{mean}$ (kN)	0.2705	0.2845	0.2947	0.5040	0.8188
Maximum absolute error ${\left|e\right|}_{\max}$ (kN)	0.9635	1.1992	1.1585	1.6764	2.7220
Goodness of fit $R^{2}$	0.9904	0.9903	0.9898	0.9822	0.9705

**Table 5:** The values of the parameters for the modified polynomial model under a strain rate of 1 mm/s.

No.	*n*	*m*	*a*_1_	*a*_2_	*a*_3_	*a*_4_	*a*_5_
1#	1.559	1.3	−7.095	−17.270	−2.966	7.961	−1.561
2#	1.582	1.3	−10.480	−8.339	−13.888	14.152	−2.898
3#	0.4405	2.7	−2.215	41.363	−47.008	24.473	−4.123
4#	0.7647	2.0	−18.071	97.066	−140.280	83.238	−16.947

**Table 6:** Comparison of simulation fitting errors under strain rate of 1 mm/s.

Evaluation Indicator	Bearing Specimen			
	1#	2#	3#	4#
Mean square error $e_{MSE}$ (kN)	0.0834	0.2371	0.1304	0.4134
Mean absolute error ${\left|e\right|}_{mean}$ (kN)	0.2134	0.3895	0.2816	0.5215
Maximum absolute error ${\left|e\right|}_{\max}$ (kN)	1.8563	1.5747	1.1458	2.0148
Goodness of fit $R^{2}$	0.9920	0.9865	0.9902	0.9822

**Table 7:** Vertical mechanical performance test conditions for thick laminated rubber bearing.

No.	Test Contact Pressure (MPa)	Standard Load (kN)	Vertical Load (kN)
1	4 ± 10%	6760	6084–7436
2	4 ± 30%	6760	4732–8788
3	6 ± 10%	10,140	9126–11,154
4	6 ± 30%	10,140	7098–13,182

**Table 8:** Modified polynomial model parameters for the thick laminated rubber bearing.

Test Contact Pressure (MPa)	*n*	*m*	*a*_1_	*a*_2_	*a*_3_	*a*_4_	*a*_5_
4 MPa ± 10%	0.155	20	647.073	−1573.833	3430.332	525.548	−811.441
4 MPa ± 30%	0.263	5	1370.000	−7879.155	13,115.430	−6316.940	973.649
6 MPa ± 10%	0.190	15	−1341.604	3184.703	−891.596	668.359	−200.585
6 MPa ± 30%	0.137	15	−3076.546	7914.805	−4848.866	1704.306	−236.524

**Table 9:** Simulation fitting errors for the thick laminated rubber bearing.

Evaluation Indicator	Test Contact Pressure (MPa)			
	4 MPa ± 10%	4 MPa ± 30%	6 MPa ± 10%	6 MPa ± 30%
Mean square error $e_{MSE}$ (kN)	63.2883	138.7204	118.0534	246.2701
Mean absolute error ${\left|e\right|}_{mean}$ (kN)	52.0690	115.7930	97.9665	209.1763
Maximum absolute error ${\left|e\right|}_{\max}$ (kN)	233.0612	412.3403	408.9223	538.8545
Goodness of fit $R^{2}$	0.9901	0.9788	0.9877	0.9748

## Data Availability

The original contributions presented in this study are included in the article. Further inquiries can be directed to the corresponding author.
